# Universal lasing condition

**DOI:** 10.1038/s41598-021-83701-3

**Published:** 2021-02-18

**Authors:** Ilya V. Doronin, Alexander A. Zyablovsky, Evgeny S. Andrianov, Alexander A. Pukhov, Yurii E. Lozovik, Alexey P. Vinogradov

**Affiliations:** 1 Dukhov Research Institute of Automatics (VNIIA), 22 Sushchevskaya, Moscow, 127055 Russia; 2 Moscow Institute of Physics and Technology, 9 Institutskiy pereulok, Moscow, 141700 Russia; 3 Institute for Theoretical and Applied Electromagnetics, 13 Izhorskaya, Moscow, 125412 Russia; 4 Institute of Spectroscopy Russian Academy of Sciences, 5 Fizicheskaya, Troitsk, Moscow, 108840 Russia

**Keywords:** Lasers, LEDs and light sources, Quantum optics

## Abstract

Usually, the cavity is considered an intrinsic part of laser design to enable coherent emission. For different types of cavities, it is assumed that the light coherence is achieved by different ways. We show that regardless of the type of cavity, the lasing condition is universal and is determined by the ratio of the width of the atomic spectrum to the product of the number of atoms and the spontaneous radiation rate in the laser structure. We demonstrate that cavity does not play a crucial role in lasing since it merely decreases the threshold by increasing the photon emission rate thanks to the Purcell effect. A threshold reduction can be achieved in a cavity-free structure by tuning the local density of states of the electromagnetic field. This paves the way for the design of laser devices based on cavity-free systems.

## Introduction

The invention of lasers was one of the key achievements in physics^1^. A conventional laser consists of two main components: a cavity and a pumped active medium^2,3^. The active medium plays the role of an amplifier, while the cavity provides positive feedback, and together these form a coherent light generator^2,3^.

Initially, lasers were perceived as a combination of an amplifying medium and reflective boundaries (Fabry–Perot lasers)^3^. To describe their operation, a simple concept of light travelling along a closed path through an amplifying medium was applied^3^. According to this view^3^, lasing occurs firstly when the frequency of the electromagnetic (EM) wave is close to the transition frequency between the working levels of the active medium, and, secondly, when the amplification of the EM field by the active medium exceeds the total losses due to radiation and dissipation inside the structure of the laser^3,4^.

Subsequent developments led to the creation of new lasers, to which the concept of light travelling through amplifying medium inside the cavity could hardly be applied. One of these is the random laser^5–7^, the operation of which has been the subject of various studies (see e.g.^8,9^). In these lasers, localized states in the disordered medium play the role of laser modes. These modes have a complicated distribution of the EM field that depends on the population inversion of the active medium^9^. Another example is the spaser (plasmonic nanolaser)^10–13^, in which the EM field modes are localized at plasmonic nanoparticles. Furthermore, it has also recently been demonstrated that the generation of coherent light can occur even in cavity-free systems^14–16^. In^14,15^, it was shown that lasing can take place in a cavity-free system based on a multilayer plasmonic waveguide. The parameters of the layers are chosen to minimize the group velocity at the transition frequency of active atoms. This decrease in the group velocity results in a stopped-light lasing in the cavity-free system^14,15^. Moreover, at sufficiently high gain, lasing can even take place in a cavity-free system without a waveguide^16^. In this system, the frequency pulling caused by the nonlinear interaction between the free-space modes and the active medium leads to the formation of a localized special mode composed of a large number of free-space non-localized modes^16,17^. At a sufficiently high pump rate, lasing starts in this special mode. Thus, even without a cavity, lasing can take place^14,16,17^. In these cases, simple evaluation of the laser threshold mentioned above does not apply, and direct numerical simulations have been used to find the threshold. In this context, the question of a lasing condition that does not depend on the type of laser is important.

In this paper, we show that both cavity and cavity-free lasers can be described in the unified framework. We demonstrate that regardless of the type of cavity, the threshold population inversion in the active medium is determined by the equation $D_0^{th} = {\gamma _\sigma }/\left( {N{\gamma _{sp}}} \right)$, where ${\gamma _\sigma }$ is the linewidth of an atom, ${\gamma _{sp}}$ is the spontaneous emission rate of an atom in a given cavity, and *N* is the number of atoms. The value of ${\gamma _\sigma }$ is determined by the dephasing processes in the active medium, such as phonon scattering, whereas $N{\gamma _{sp}}$ determines the total emission rate of photons. Lasing starts when this emission rate exceeds the dephasing rate. That is, lasing takes place when, on average, the system emits more than one photon during the dephasing time.

It follows from the obtained condition that the lasing threshold can be reduced by increasing the Purcell factor (i.e., the photon emission rate) of the system containing the active medium. In conventional lasers, this is accomplished by adding the resonator. However, this can be achieved by a structure without cavity^14,15^. Thus, the resonator is not essential for lasing and only serves as a way to decrease the lasing threshold.

## Model

We consider lasers with active medium consisting of *N* two-level atoms, placed within an arbitrary system of finite size. To describe this system we use the scheme suggested in^18^ (see also^16,17,19^). We first place the system in a finite three-dimensional (3D) box with size *L*. We introduce an artificial relaxation rate $\gamma _n$ to each mode of the EM field, such that ${\gamma _n} \gg c/L$, where *c* is speed of light. The presence of artificial losses prevents the influence of radiation reflected from the box boundaries on the behavior of laser system. We then write Maxwell–Bloch equations for the active atoms and the modes of the EM field in this finite box, and find a stationary nontrivial solution^2,3,20^. Finally, we move to the limit of infinite box size and zero artificial losses in the box to obtain the lasing conditions (see “Lasing conditions”). In this limit, the artificial losses do not affect the lasing threshold and, for simplicity, we assume that for all modes ${\gamma _n} = {\gamma _a}$. The Maxwell–Bloch equations describing active atoms and modes of EM field take the form:

1 $$\begin{aligned} d{a_n}/dt= & {} - \left( {{\gamma _a} + i{\omega _n}} \right) {a_n} - i\sum \limits _m {\Omega _{nm}^ * {\sigma _m}} + F_n^a\left( t \right) \end{aligned}$$

2 $$\begin{aligned} d{\sigma _m}/dt= & {} - \left( {{\gamma _\sigma } + i{\omega _{TLS}}} \right) {\sigma _m} + i{D_m}\sum \limits _n {{\Omega _{nm}}{a_n}} + F_m^\sigma \left( t \right) \end{aligned}$$

3 $$\begin{aligned} d{D_m}/dt= & {} \left( {\gamma _m^{pump} - {\gamma _D}} \right) - \left( {\gamma _m^{pump} + {\gamma _D}} \right) {D_m} + 2i\sum \limits _m {\left( {\Omega _{nm}^ * a_n^ * {\sigma _m} - {\Omega _{nm}}{a_n}\sigma _m^*} \right) } \end{aligned}$$

Here ${a_n}$ is the complex amplitude of the nth mode of the EM field in the finite box with eigenfrequency ${\omega _n}$ and loss rate ${\gamma _a}$. ${\sigma _m}$ is the complex polarization of the mth atom of the active medium. ${\omega _{TLS}}$ is the transition frequency of active atoms, and ${\gamma _\sigma }$ is the relaxation rate of the polarization of the atom (i.e. the linewidth of the atom). The relaxation of polarization is mainly caused by dephasing processes such as the interactions between atoms and the phonons inside the active medium^21,22^, and is thus determined by the properties of the active medium. ${D_m}$ is the population inversion of the mth active atom. ${\gamma _D}$ and $\gamma _m^{pump}$ are the population inversion decay and pump rates of the mth active atom, respectively. ${\Omega _{nm}} = - {{\mathbf{d}}_{eg}} \cdot {{\mathbf{E}}_n}({{\mathbf{x}}_m})/\hbar$ is the interaction constant between the dipole moment ${{\mathbf{d}}_{eg}}$ of active atom placed at the point ${{\mathbf{x}}_m}$ and the electric field per one photon ${{\mathbf{E}}_n}({{\mathbf{x}}_m})$ of nth mode^2^. The interaction between the EM field and active medium is described in the dipole approximation. That is, we neglect the interaction term $V \simeq {e^2}{A^2}/\left( {2m{c^2}} \right)$ proportional to the square of the vector potential since it is small compared to electro-dipole interaction when system outside the ultrastrong-coupling regime^23^. $F_n^a\left( t \right)$ and $F_m^\sigma \left( t \right)$ are noise terms, which connect with the relaxation rates in Eqs. (1)–(3) via the fluctuation–dissipation theorem^21^. We also introduce the notation ${D_{0m}} = \left( {\gamma _m^{pump} - {\gamma _D}} \right) /\left( {\gamma _m^{pump} + {\gamma _D}} \right)$ for the stationary value of the population inversion of the mth atom at zero amplitudes of the modes.

Note that the general form of the Maxwell–Bloch equations (1)–(3) does not depend on the specific structure of the laser. The distribution of the EM field in the eigenmodes of the total system containing all necessary information about the properties of the cavity is included in Eqs. (1)–(3) by means of the coupling constants ${\Omega _{nm}}$^2,20,21^. The active medium is described as an array of two-level atoms. Three- and four-level active media can often be considered as two-level media, if one eliminates degrees of freedom corresponding to the third or fourth level^20^. Equations (1) and (2) have the same form for three and four-level active media^20^. At the same time, the coefficients in the Eq. (3) for population inversion of active atoms depend on the specific type of active medium^20^ (Eq. 3 is written for a three-level active medium). However, below we are interested in the threshold population inversion, which is determined from the linearized version of Eqs. (1)–(2) (see section “Lasing conditions”) and does not depend on the specific form of Eq. (3). For this reason, the coefficients in Eq. (3) do not matter for us. Thus, Eqs. (1)–(3) describe all types of laser structure within a unified framework.

In terms of the Maxwell–Bloch equations (1)–(3) without noises^2,20^, below a certain pump value, the stationary amplitude of electric field is zero. Above this value, the amplitude of electric field becomes nonzero. Therefore, this value is referred to as the lasing threshold. Since the Maxwell–Bloch equations (1)–(3) without noise terms are deterministic, the EM field calculated by these equations has zero linewidth and its second order coherence function ${g^{\left( 2 \right) }}\left( 0 \right)$ is equal to 1. Thus, above the lasing threshold (determined as pump rate at which a non-zero amplitude is achieved) the radiation is immediately fully coherent as one would expect from this model. Taking into account the noise terms in Eqs. (1)–(3), the amplitude of the electric field is nonzero both below and above the lasing threshold. As has been shown in^2,20^ the noise terms in the Maxwell–Bloch equations (1)–(3) allow one to describe phenomena associated with spontaneous emission^2,20^. Noise leads to a phase disturbance of the EM field and the atomic polarization, which results in nonzero linewidth^2,24^ and deviation of ${g^{\left( 2 \right) }}\left( 0 \right)$ from 1. Below the lasing threshold, the electric field is generated by noise and ${g^{\left( 2 \right) }}\left( 0 \right) \approx 2$ (see^19^). Above the lasing threshold, a deterministic contribution to the electric field appears. As a result, above the lasing threshold, the radiation linewidth decreases and ${g^{\left( 2 \right) }}\left( 0 \right)$ changes from 2 to 1 with an increase of the pump rate. ${g^{\left( 2 \right) }}\left( 0 \right)$ reaches 1 when the deterministic contribution to the EM field becomes much larger than the contribution of spontaneous radiation. In high-Q lasers, this occurs practically at the lasing threshold^2,24^. In low-Q lasers, there is a transition region where ${g^{\left( 2 \right) }}\left( 0 \right)$ changes smoothly from 2 to 1 (see, for example,^16^). Thus, the lasing threshold corresponds to the pumping rate at which a coherent signal originates in the system.

## Lasing conditions

Using the framework described in the previous section, we can find the lasing threshold and lasing frequencies for an arbitrary type of cavity. Remember that we consider the laser, which is placed in a 3D box with size *L*, which plays the role of the environment (see^2,16,18,19^). In Eqs. (1)–(3), several parameters depend on *L*. In particular, in 3D space, the value of the coupling constants, ${\Omega _{nm}}$, is proportional^2,21^ to ${L^{ - 3/2}}$, and the frequency interval between the two closest modes, ${\omega _{n + 1}} - {\omega _n}$, is proportional^2^ to ${L^{ - 1}}$. However, as we show below (see subsection “General lasing condition” of “Methods”) in the limit $L \rightarrow \infty$ the expression for the lasing threshold does not depend on *L* and tends to a finite value.

In addition, we emphasize the significant role of the EM field relaxation rate ${\gamma _a}$. In a finite box, perfectly reflecting boundaries cause any outgoing radiation to travel back into the system and to affect its dynamics. Since we aim to describe a cavity and an active medium located in free space, we introduce an artificial relaxation rate ${\gamma _a}$ to each mode of the EM field, such that ${\gamma _a} \gg c/L$, where *c* is speed of light (see^16,19^). This condition ensures that the effect of reflection from the boundaries on the active medium is negligible. Hence, even for finite *L*, our model is close to a system placed in free space, in which radiation leaves the system and does not return. Finally, we take the limit $L \rightarrow \infty$ and arrive at an infinite system with loss, and eliminate artificial losses by taking ${\gamma _a} \rightarrow 0$. Such a passage to the limits is known as the Limiting Absorption Principle^25–27^. In this limit we get the solution corresponding to the radiation of waves from the active medium into the external space^28^.

To obtain the lasing threshold, we search for a nontrivial solution of Eqs. (1)–(3) without noise terms. The Maxwell–Bloch equations (1)–(3) without noise have trivial solution ${a_n} = {\sigma _m} = 0$ and ${D_m} = D_{0m}$. However, this solution is unstable above lasing threshold where non-trivial solution with ${a_n} \ne 0$ and ${\sigma _m} \ne 0$ appears. Following standard procedure in laser physics^2,3^, we linearize the Maxwell–Bloch equations in the vicinity of the trivial solution. The threshold is defined as such value of population inversion at which linearized system of equations has eigenvalue with zero real part (see also^17,29^). In the general case of a laser with an extended active medium the lasing condition is determined by a homogeneous Fredholm integral equation of the second kind for the Fourier amplitudes of the averaged atomic polarization of the active medium (see “General lasing condition” of “Methods”):

4 $$\begin{aligned} \begin{array}{l} \left( {{\gamma _\sigma } + i\,({\omega _{TLS}} - {\omega _g})} \right) S({\mathbf{x}}) = {D_0}\left( {\mathbf{x}} \right) \sum \limits _\alpha {\int {\frac{{{d^3}{\mathbf{k}}}}{{{{\left( {2\pi } \right) }^3}}}} } \left[ {\frac{{{\Omega _\alpha }\left( {{\mathbf{x}},{\mathbf{k}}} \right) }}{{{\gamma _a} + i{} \,(ck - {\omega _g})}}\int {{d^3}{\mathbf{y}}\,n\left( {\mathbf{y}} \right) \,\Omega _\alpha ^ * \left( {{\mathbf{y}},{\mathbf{k}}} \right) \,S\left( {\mathbf{y}} \right) } } \right] \end{array} \end{aligned}$$

where ${\Omega _\alpha }\left( {{\mathbf{x}},{\mathbf{k}}} \right)$ is the effective interaction constant between atoms at the point ${\mathbf{x}}$ and the EM mode with wave vector ${\mathbf{k}}$; $n\left( {\mathbf{y}} \right)$ is the density of active atoms; $S({\mathbf{x}})$ is the Fourier transform of the averaged atomic polarization at the point ${\mathbf{x}}$; ${D_0}\left( {\mathbf{x}} \right)$ is the average population inversion of active atoms at the point ${\mathbf{x}}$; and ${\omega _g}$ is the generation frequency. Although this equation provides us a criterion for laser action in arbitrary medium, it is complicated to solve in the general case. We therefore simplify our model to obtain a result that has a transparent interpretation. We consider a model of a laser in which all active atoms are located at one point, ${\mathbf{x}} = 0$. The general condition (4) can then be simplified to (see “General lasing condition” of “Methods”)

5 $$\begin{aligned} D_{th} = \frac{{{\gamma _\sigma }}}{{N{\gamma _{sp}}({\omega _g})}} \end{aligned}$$

where the lasing frequency ${\omega _g}$ is determined by the expression

6 $$\begin{aligned} {\omega _g} = {\omega _{TLS}} + \frac{{{\gamma _\sigma }}}{{{\gamma _{sp}}({\omega _g})}}\Delta ({\omega _g}) \end{aligned}$$

where ${\gamma _{sp}}({\omega _g})$ is the spontaneous emission rate of atoms at the lasing frequency, and $\Delta ({\omega _g})$ is the Lamb shift in the laser structure^21^, see Eq. (24). Note that it differs from the energy level shift of atoms without pump in vacuum. Thus, the lasing threshold and the lasing frequency are determined by the spontaneous emission rate, the dephasing rate of active atoms (the linewidth of atoms), and the frequency shift in the laser structure. These expressions are obtained from the general lasing condition (4) under the assumption that the active medium occupies subwavelength volume. This approximation is valid, for example, for the plasmonic nanolaser^10–12,30^.

We emphasize that ${\gamma _{sp}}({\omega _g})$ in the expression for the lasing threshold (5) arises from a combination of parameters included in the integral equation (4). This quantity characterizes the magnitude of the interaction of the EM field with the active medium placed inside the laser structure. ${\gamma _{sp}}({\omega _g})$ is proportional to the local density of states (LDOS) of the electromagnetic modes at the location of the atoms (Purcell effect)^31^. The ratio of the spontaneous emission rate, ${\gamma _{sp}}$, to that in free space, $\gamma _{sp}^{vac}$, is referred to as the Purcell factor ${F_P}$ (i.e., ${F_P} = {\gamma _{sp}}/\gamma _{sp}^{vac}$)^31^. It is equal to the ratio of the LDOS in a given system to the LDOS in free space. According to Eq. (5), the lasing threshold is inversely proportional to the Purcell factor. The other quantities in Eq. (5) are determined by the properties of the active medium, and do not depend on the EM mode structure of the system. Thus, the influence of the system in which the atoms are placed is reduced to the modification of the spontaneous emission rate due to the Purcell effect.

## Influence of the LDOS on the lasing threshold

### Lasing in free space

An active medium in free space can lase even without an external cavity, provided that the optical gain in the medium is sufficient^16^. To illustrate this statement, we consider a layer of active atoms placed in free-space. For simplicity, we study system in one-dimensional approximation. For layer of active atoms (1D), the threshold population inversion $D_{th}$ and the lasing frequency $\omega _g$ are usually determined by the following condition^4,32^:

7 $$\begin{aligned} r{\left( {{\omega _g},{D_{th}}} \right) ^2}\exp \left( {2\,i\,\frac{{{\omega _g}}}{c}\,\sqrt{{\varepsilon _{gain}}\left( {{\omega _g},{D_{th}}} \right) } \,l} \right) = 1 \end{aligned}$$

Here *l* is the length of the active region; ${\varepsilon _{gain}}\left( {\omega ,D} \right) = 1 - \alpha \,n\,D/\left( {{\omega _{TLS}} - \omega - i\,{\gamma _\sigma }} \right)$ is the dielectric constant of active medium consisting of two-level atoms, where $\alpha = 4\pi {\left| {{{\mathbf{d}}_{eg}}} \right| ^2}/\hbar$; ${{\mathbf{d}}_{eg}}$ and *n* are the dipole moment of atoms and the atomic concentration, respectively^4^. $r\left( {\omega ,D} \right) = \left( {\sqrt{{\varepsilon _{gain}}\left( {\omega ,D} \right) } - 1} \right) /\left( {\sqrt{{\varepsilon _{gain}}\left( {\omega ,D} \right) } + 1} \right)$ is the reflection coefficient from the boundary between the active medium and the free space^4^. The dielectric constant of the active medium can be expressed through the effective interaction constants between atoms and the EM mode ${\Omega _\alpha }\left( {{\mathbf{x}},{\mathbf{k}}} \right)$ (see, for example,^33,34^), which are included in the integral equation (4). The coordinate dependencies of ${\Omega _\alpha }\left( {{\mathbf{x}},{\mathbf{k}}} \right)$ are determined by the eigenmodes of the system ${{\mathbf{E}}_n}\left( x \right)$, which, for a one-dimensional empty box, are $\sim \cos \left( {kx} \right)$ and $\sim \sin \left( {kx} \right)$.

**Figure 1:**
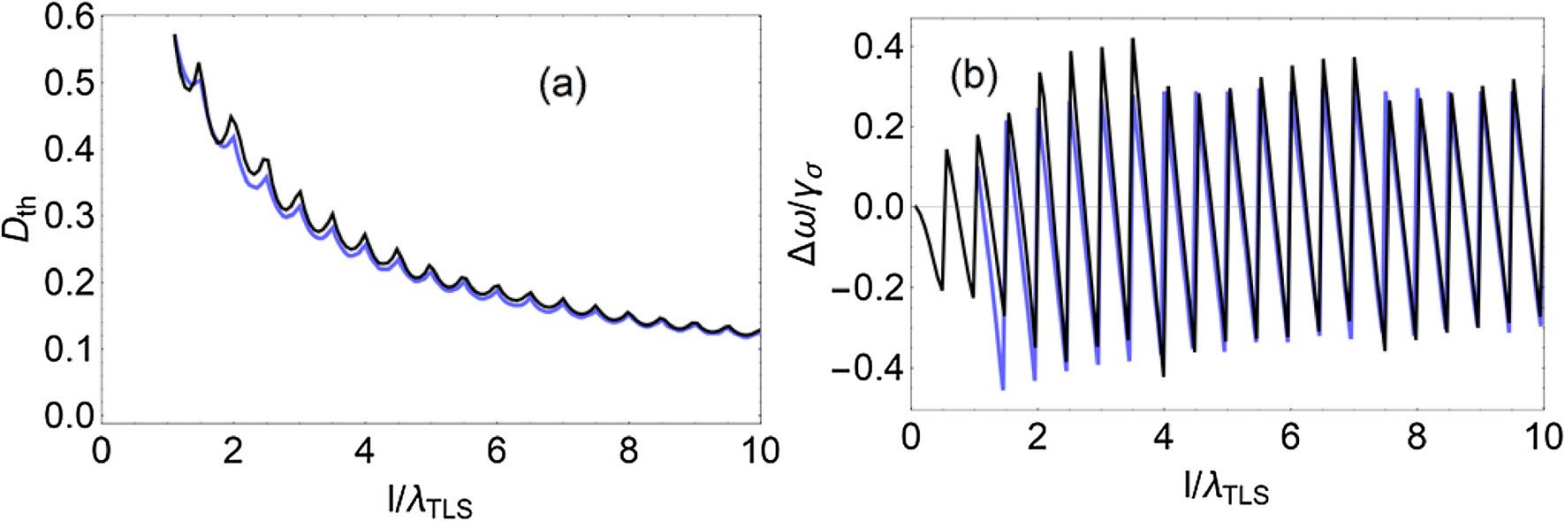
Dependence of the threshold population inversion $D_{th}$ (**a**) and the lasing frequency $\omega _g$ (**b**) on the length of the active region *l* at a constant concentration of atoms *n*. The black line is calculated by the Eq. (4); the blue line is calculated by the Eq. (7). The effective interaction constants between the atoms and the EM mode ${\Omega _\alpha }\left( {x,k} \right) = {\Omega _0}\cos \left( {k\,x} \right)$ and ${\Omega _\alpha }\left( {x,k} \right) = {\Omega _0}\sin \left( {k\,x} \right)$, where $k = \omega /c$. The following values of the system parameters are used: ${\Omega _0} = {10^{ - 6}}{\omega _{TLS}}$, ${\gamma _\sigma } = 5 \times {10^{ - 5}}\,{\omega _{TLS}}$, ${\gamma _D} = 1 \times {10^{ - 6}}\,{\omega _{TLS}}$, ${n_c} = 1.25 \times {10^4}\,\lambda _{TLS}^{ - 1}$, where ${\lambda _{TLS}} = 2\pi c/{\omega _{TLS}}$.

The expression (7) for lasing threshold is written under the assumption that lasing occurs at one of mode of Fabry-Perot resonator formed by the finite layer of the active medium. The lasing threshold, determined by formula (7), is close to the exact value of the lasing threshold, calculated using the integral equation (4). To illustrate this statement, we consider the dependence of the lasing threshold $D_{th}$ on the length of the active medium *l* assuming the concentration of active atoms is constant. When we decrease the length of the active medium then the lasing threshold increases (Fig. 1a). In addition, the lasing frequency periodically varies with the change in length (Fig. 1b), which leads to periodic changes in the threshold population inversion (Fig. 1a). The period of changes is equal to half the wavelength. The described behavior is predicted by both Eqs. (4) and (7).

Thus, we conclude that the active medium in free space can lase. Lasing occurs when the radiation losses are compensated by the amplification of light in the active medium. Note that due to the spontaneous emission, an amplified spontaneous emission (ASE) takes place in the active medium below the lasing threshold^19^. Spontaneous emission of excited atoms induces electromagnetic radiation, which amplifies passing through the active medium, but the gain coefficient is insufficient for lasing. It is known that ASE system can demonstrate the threshold dependence of the output radiation on the pump rate^19^. However, in contrast to laser radiation, the second order coherence function, ${g^{\left( 2 \right) }}\left( 0 \right)$, of amplified spontaneous radiation is equal to 2^16,19^.

The well-known example of cavity-free lasers is astrophysical lasers, which form in clouds of interstellar gas^35–37^ and in the atmospheres of planets^38,39^. Depending on the gain and length of the active medium, these structures can be either ASE sources^40^ or lasers^37,41^.

Active atoms occupying a subwavelength volume in free space presents another interesting type of cavity-free lasers. Such a system is typically considered to be incapable of lasing since there is no laser mode in the subwavelength volume. However, the interaction of active atoms with a continuum of free space modes can lead to the formation of a laser mode in such a system^17^. The lasing threshold for this system is determined by Eq. (5). Assuming for evaluation that ${\gamma _\sigma } \sim {10^{13}}\,{s^{ - 1}}$, ${\gamma _{sp}} \sim {10^9}\,{s^{ - 1 }}$, $\lambda \sim 500$ nm^42^ and the subwavelength volume occupied by the active atoms is $V \sim {10^{ - 3}}{\lambda ^3}$, we find that at the lasing threshold, the necessary number of active atoms is ${N} \sim {10^4}$ and the concentration of active atoms is ${n_c} = {N}/V \sim {10^{20}}\,{{\text {cm}}^{ - 3}}$. These estimates suggest that the cavity-free laser requires a gain medium with a high concentration of active atoms, which makes it difficult to create.

### Using a resonator to reduce the lasing threshold

Lasing in the free space can be occur only at high gain and length of the active medium. Usually, cavity is used to decrease the lasing threshold. Adding a resonator leads to a change in the local density of states of the EM field (LDOS). As shown in^2^, a single-mode cavity can be considered as a structure with the density of states $\rho (\omega ) = {\gamma _{CM}}/\pi \left[ {\gamma _{CM}^2 + {{(\omega - {\omega _{CM}})}^2}} \right]$. The Purcell factor of this single-mode cavity is

8 $$\begin{aligned} {F_P} = \frac{3}{{4\,{\pi ^2}}}\frac{{{\lambda ^3}}}{V}\frac{{{\gamma _{CM}}\,{\omega _{CM}}}}{{\gamma _{CM}^2 + {{(\omega - {\omega _{CM}})}^2}}} \end{aligned}$$

Here ${{\omega _{CM}}}$ and ${{\gamma _{CM}}}$ are the frequency and the relaxation rate of the cavity mode; $\lambda$ is the wavelength of cavity eigenmode; *V* is the cavity volume. In the resonance case, ${F_P} = \frac{3}{{4\,{\pi ^2}}}\frac{{{\lambda ^3}}}{V}\frac{{{\omega _{CM}}}}{{{\gamma _{CM}}}} = \frac{3}{{4\,{\pi ^2}}}\frac{{{\lambda ^3}}}{V}Q$, where $Q = {\omega _{CM}}/{\gamma _{CM}}$ is quality factor.

The lasing threshold (5) is inversely proportional to the Purcell factor, $F_P$. There are two ways to increase the Purcell factor of cavity (8). The first way is to increase the Q-factor of the resonator ($Q = {\omega _{CM}}/{\gamma _{CM}}$). This approach to lowering the laser threshold is the most common in laser physics. Note that when $Q> > 1$ the Purcell factor has a sharp maximum at the frequency of the cavity mode, ${{\omega _{CM}}}$, and so the lasing frequency is close to the frequency of cavity mode.

The second way is to decrease the cavity volume *V*. This approach is used, for example, in the plasmonic nanolaser lasers (spasers)^10^, where the EM field is localized in subwavelength volume ($V < {\lambda ^3}$). Note that for lasers with $V> > {\lambda ^3}$ the Purcell factor (8) can be less than one. However, a decrease in the Purcell factor (${F_P} \sim {V^{ - 1}}$) can be compensated by an increase in the number of active atoms, which is usually proportional to the cavity volume ($N \sim V$).

The expressions for the lasing threshold (5) and the lasing frequency (6) agree with the well-known expressions for the lasing threshold and the lasing frequency of the single-mode laser^2^:

9 $$\begin{aligned} {D_{th}}= & {} \frac{{{\gamma _{CM}}{\gamma _\sigma }}}{{N\Omega _{cav}^2}}\left( {1 + \frac{{{{({\omega _{CM}} - {\omega _{g}})}^2}}}{{\gamma _{CM}^2}}} \right) \end{aligned}$$

10 $$\begin{aligned} {\omega _g}= & {} \frac{{{\gamma _{CM}}{\omega _{TLS}} + {\gamma _\sigma }{\omega _{CM}}}}{{{\gamma _\sigma } + {\gamma _{CM}}}} \end{aligned}$$

where ${\Omega _{cav}}$ is a coupling constant of the EM field mode with the active medium. Indeed, using the density of state of the EM field in the single-mode cavity $\rho (\omega ) = {\gamma _{CM}}/\pi \left[ {\gamma _{CM}^2 + {{(\omega - {\omega _{CM}})}^2}} \right]$^2^, we obtain the expressions (9) and (10) from the Eqs. (5) and (6) (for details, see “Single-mode laser” of “Methods”). Thus, the solution of the classical problem of a single-mode laser is obtained by solving Eqs. (5) and (6).

Note that the presence of the cavity leads to a change in the coupling constant between the EM field and the active medium caused by the change in the LDOS. That results in a change in the spontaneous emission rate of active atoms and in a change in the lasing threshold.

### Reducing the lasing threshold using cavity-free structures

**Figure 2:**
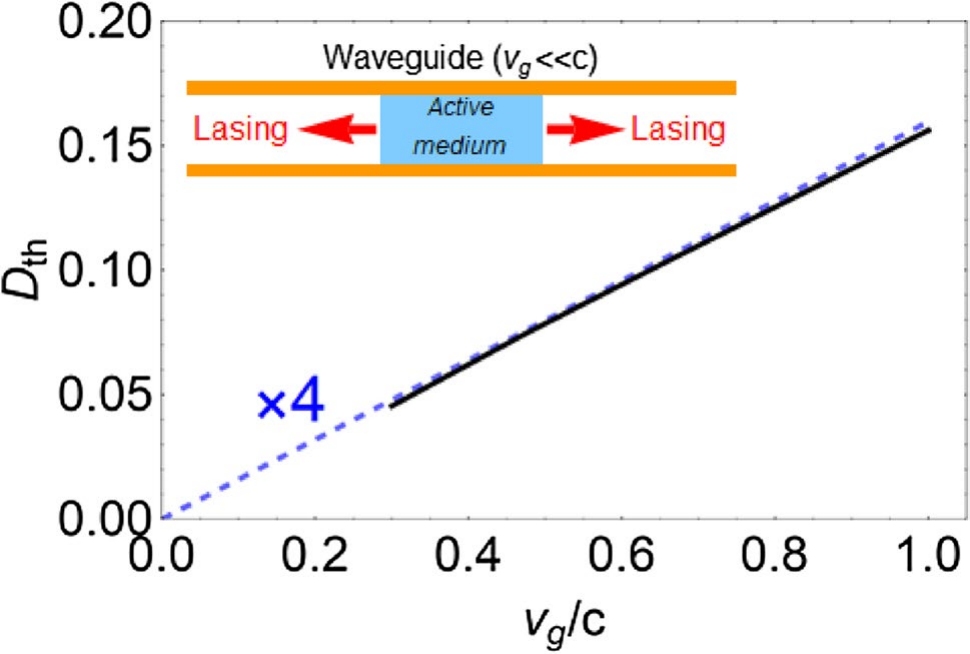
Dependence of the threshold population inversion ${D_{th}}$ on the group velocity in waveguide. The black solid line is calculated by Eq. (4) for the case of an extended active medium ($l = 8 \lambda$). The blue dashed line is calculated by Eq. (5) for the case a subwavelength active medium. Inset: sketch of laser based on waveguide with low group velocity.

It is not necessary to use a resonator to decrease the lasing threshold. The threshold can be decreased by using cavity-free structures with Purcell factor ${F_P} > 1$. Use of such structures enables to increase the interaction of light with the active medium that in turn leads to a decrease in the lasing threshold. Waveguides with a group velocity ${v_g}< < c$ (see inset in Fig. 2), e.g., line-defect waveguides in photonic crystal^43–47^ or plasmonic waveguides^14,15^ can play the role of such structure. The LDOS in these structures is increased by a factor of $c/{v_g}$, leading to the enhancement of light-matter interaction^44,45,47^. This effect enables to use such waveguides to decrease the lasing threshold. For an example, in^14,15^, it has been demonstrated that lasing takes place in a planar waveguide filled with the active medium. The authors achieved a near-zero group velocity for the optical waves (${v_g} \sim {10^{ - 4}}\,c$) by adjusting the dimensions of the waveguide. In turn, this leads to a growth of the spontaneous emission rate of active atoms and, according to Eq. (5), to a decrease in the lasing threshold (see Fig. 2).

In Ref.^14^ the active medium occupying the subwavelength volume in the plasmonic waveguide has been considered. However, the conclusion that decrease the lasing threshold when a group velocity is reduced remains valid for an extended active medium. We consider the active layer with length $l> > \lambda$ placed in a waveguide with the group velocity ${v_g} = d\omega /dk$ (see inset in Fig. 2). Although there are no mirrors to form the cavity, lasing occurs in this structure. Reduction of the group velocity ${v_g}$ leads to a decrease in the lasing threshold, ${D_{th}} \sim {v_g/c}$, (see Fig. 2), which is calculated by Eq. (4). This is due to an intensification in the interaction of light with an active medium with an increase in the LDOS. This clarifies the mechanism of lasing based on stopped light.

Thus, the increase of LDOS leads to the increase of Purcell factor and the strength of light-active atoms interaction. In turn, this leads to a decrease in the lasing threshold of lasers with both subwavelength (see Eq. (5)) and distributed active media (see Eq. 4). The specific type of structure, by means of which the LDOS increases, does not affect the lasing threshold. That is, the lasing threshold can be decreased by using cavity-free structure.

## Discussion

The forms of Eqs. (5) and (6) do not depend explicitly on the properties of the system, and this indicates the existence of a unified mechanism for lasing in lasers with arbitrary types of cavity, and even without a cavity. The expression for the lasing threshold in Eq. (5) can be rewritten as $ND_0^{th}{\gamma _{sp}} > {\gamma _\sigma }$. The product of $ND_0^{th}$ is equal to the difference between the numbers of atoms in the excited and ground states. In an EM field, atoms radiate at a rate proportional to the number of atoms in the excited state, $N\left( {D_0^{th} + 1} \right) /2$. Simultaneously, atoms absorb radiation at a rate proportional to the number of atoms in the ground state, $N\left( {1 - D_0^{th}} \right) /2$. The difference between these quantities, $ND_0^{th}$, determines the net emission rate caused by the radiated field^10^. In this way, the product $ND_0^{th}{\gamma _{sp}} = {\gamma _{rad}}$ may be interpreted as the rate of photon emission of atoms induced by a previously emitted photon. The lasing takes place when the radiation rate ${\gamma _{rad}}$ exceeds the dephasing rate ${\gamma _\sigma }$ (the linewidth of the atom) i.e., when the system emits more than one photon during the dephasing time. In this case, the radiation is coherent.

The origination process of coherence can be described in the following way. Each atom in the ensemble is affected by emission from all other atoms. This emission acts as an external driving force, causing the atom to oscillate at a unified frequency. On the other hand, dephasing processes occurring due to interaction with the environment (for details, see^22,48^) cause the phase of oscillations to be disturbed. If the action of all atoms upon each single atom can overcome dephasing, atoms start to synchronize and emit coherently with each other, which leads to a substantial increase in the atom-field interaction and lasing. To overcome dephasing, the radiation rate ${\gamma _{rad}}$ must exceed the dephasing rate ${\gamma _\sigma }$.

It should be noted that according to the lasing condition in Eq. (5), there are two ways to decrease the lasing threshold. The first is to increase the Purcell factor in the system (i.e., the photon emission rate) containing the active medium. This approach is applied in conventional lasers by using the cavity. The second is based on the use of an active medium with narrow linewidth, i.e., decrease the dephasing rate. This is utilized in superradiance lasers^49–53^.

## Conclusions

In conclusion, we show that both cavity and cavity-free lasers can be described in the unified framework. For the case of an active medium localized in a subwavelength volume, we demonstrate that regardless of the type of cavity, the threshold population inversion in the active medium is determined by the equation $D_0^{th} = {\gamma _\sigma }/N{\gamma _{sp}}$, where ${\gamma _\sigma }$ is the linewidth of the atoms, ${\gamma _{sp}}$ is the spontaneous emission rate of the atoms in the laser structure, and *N* is the number of active atoms. The value of ${\gamma _\sigma }$ is determined by the dephasing processes in the active medium, such as elastic phonon scattering, whereas ${N\gamma _{sp}}$ determines the total emission rate of photons. Lasing starts when this emission rate exceeds the dephasing rate.

It follows from the obtained condition that the lasing threshold can be reduced by increasing the Purcell factor in the system (i.e., the photon emission rate) containing the active medium. In conventional lasers, the resonator enhances the photon emission rate. However, this can be achieved by a structure without cavity. Thus, the resonator is not essential for lasing and only serves as a way to decrease the lasing threshold. This result emphasizes the universal mechanism of lasing in lasers with and without cavity, which is the mutual effect of active atoms on each other leading to the buildup of coherence.

We believe that our result provides insight into the operation of lasers and is relevant to both theory and practice.

## Methods

### General lasing condition

In this section, we derive the condition for lasing in the case of a distributed active medium. We divide the active region into subwavelength cells, and write equations for the average polarization ${{\tilde{\sigma }} _m}$ and population inversion ${{{\tilde{D}}}_m}$ for each cell:

11 $$\begin{aligned} d{a_n}/dt= & {} - \left( {{\gamma _a} + i{\omega _n}} \right) {a_n} - i\sum \limits _m {\Omega _{nm}^*{N_m}{{{\tilde{\sigma }} }_m}} \end{aligned}$$

12 $$\begin{aligned} d{{\tilde{\sigma }} _m}/dt= & {} - \left( {{\gamma _\sigma } + i{\omega _{TLS}}} \right) {{\tilde{\sigma }} _m} + i{{{\tilde{D}}}_m}\sum \limits _n {{\Omega _{nm}}{a_n}} \end{aligned}$$

13 $$\begin{aligned} d{{{\tilde{D}}}_m}/dt= & {} \left( {\gamma _m^{pump} - {\gamma _D}} \right) - \left( {\gamma _m^{pump} + {\gamma _D}} \right) {{{\tilde{D}}}_m} + 2i\sum \limits _n {\left( {\Omega _{nm}^*a_n^*{{{\tilde{\sigma }} }_m} - {\Omega _{nm}}{a_n}{\tilde{\sigma }} _m^*} \right) } \end{aligned}$$

where ${N_m}$ is the number of active atoms in the mth cell. We then write the Fourier transform of the linearized Eqs. (11)–(12):

14 $$\begin{aligned} - i\,{\omega _g}{A_n}= & {} - \left( {{\gamma _a} + i{\omega _n}} \right) {A_n} - i\sum \limits _l {\Omega _{nl}^*{N_l}{S_l}} ,\,\,\,n = 1,\ldots \end{aligned}$$

15 $$\begin{aligned} - i\,{\omega _g}{S_m}= & {} - \left( {{\gamma _\sigma } + i{\omega _{TLS}}} \right) {S_m} + i\,{D_{m0}}\sum \limits _j {{\Omega _{jm}}{A_j}} ,\,\,\,m = 1,\ldots \end{aligned}$$

Here, ${A_n},\,\,\,n = 1,\ldots$ are the Fourier amplitudes of the finite box modes, and ${S_m},\,\,\,m = 1,\ldots$ are the Fourier amplitudes of the average atomic polarization in each cell of the active medium. ${D_{m0}}$ is the average population inversion. We then eliminate variables describing the EM field, ${A_n},\,\,\,n = 1,\ldots$ and obtain

16 $$\begin{aligned} - i\,{\omega _g}{S_m} = - \left( {{\gamma _\sigma } + i{\omega _{TLS}}} \right) {S_m} + \,{D_{m0}}\sum \limits _j {\frac{{{\Omega _{jm}}\sum \nolimits _l {\Omega _{jl}^*{N_l}{S_l}} }}{{{\gamma _a} + i\left( {{\omega _j} - {\omega _g}} \right) }}} ,\,\,\,m = 1,\ldots \end{aligned}$$

The next step is to move to the limit of an infinitely large box with infinitely small cells. First, in the limit of infinitely small cells, the *l* and *m* indices are transformed into continuous coordinates, and the equations (16) take the form

17 $$\begin{aligned} S\left( {\mathbf{x}} \right) = \int {{d^3}{\mathbf{y}}\left[ {\sum \limits _j^{{\mathrm{modes}}} {\frac{{\Omega _j^*\left( {\mathbf{y}} \right) {\Omega _j}\left( {\mathbf{x}} \right) }}{{\left( {{\gamma _\sigma } + i\left( {{\omega _{TLS}} - {\omega _g}} \right) } \right) \left( {{\gamma _a} + i\left( {{\omega _j} - {\omega _g}} \right) } \right) }}n\left( {\mathbf{y}} \right) {D_0}\left( {\mathbf{x}} \right) } } \right] } \,S\left( {\mathbf{y}} \right) \end{aligned}$$

where $n\left( {\mathbf{y}} \right)$ is the atomic concentration at point ${\mathbf{y}}$, ${D_0}\left( {\mathbf{x}} \right)$ is the dependence of the population inversion of the atom created by pumping at zero field amplitude on the coordinate; and ${\Omega _j}\left( {\mathbf{x}} \right)$ is the coupling constant between the *j*th mode of the EM field and the active atoms at point ${\mathbf{x}}$. Then, in the limit of an infinitely large box, the discrete variables ${\omega _j}$ are transformed into a continuous variable *k*, and the sum $\sum \nolimits _j {}$ is replaced by the integral $\frac{V}{{{{\left( {2\pi } \right) }^3}}}\sum \limits _\alpha {\int {{d^3}{\mathbf{k}}} }$, where $\alpha$ is the index denoting a particular polarization of light (not to be confused with the atomic polarization, $\sigma$). The resulting integral equation takes the form:

18 $$\begin{aligned} \left( {{\gamma _\sigma } + i\left( {{\omega _{TLS}} - {\omega _g}} \right) } \right) S\left( {\mathbf{x}} \right) = {D_0}\left( {\mathbf{x}} \right) \sum \limits _\alpha {\int {\frac{{{d^3}{\mathbf{k}}}}{{{{\left( {2\pi } \right) }^3}}}} } \left[ {\frac{{{\Omega _\alpha }\left( {{\mathbf{x}},{\mathbf{k}}} \right) }}{{\left( {{\gamma _a} + i\left( {ck - {\omega _g}} \right) } \right) }}\int {{d^3}{\mathbf{y}}\,} n\left( {\mathbf{y}} \right) \Omega _\alpha ^*\left( {{\mathbf{y}},{\mathbf{k}}} \right) S\left( {\mathbf{y}} \right) } \right] \end{aligned}$$

Here, in the limit $L \rightarrow \infty$ we denote ${\Omega _j}\left( {\mathbf{x}} \right) {L^{3/2}} \rightarrow {\Omega _\alpha }\left( {{\mathbf{x}},{\mathbf{k}}} \right)$. ${D_0}\left( {\mathbf{x}} \right)$ and ${\omega _g}$, at which there is the nontrivial solution of Eq. (18), determine the threshold population inversion $D_{th}$ and the lasing frequency.

Although Eq. (18) provides us with a criterion for lasing action in an arbitrary medium, it is complicated to solve in the general case. In some simple cases, however, the solution can be readily obtained, e.g. for $n\left( {\mathbf{y}} \right) = N\delta \left( {\mathbf{y}} \right)$, which corresponds to an active medium localized within a small subwavelength volume. After simple algebra, we obtain:

19 $$\begin{aligned} \left( {{\gamma _\sigma } + i\left( {{\omega _{TLS}} - {\omega _g}} \right) } \right) S\left( 0 \right) = {D_0}\left( 0 \right) N\sum \limits _\alpha {\int {\frac{{{d^3}{\mathbf{k}}}}{{{{\left( {2\pi } \right) }^3}}}} } \left[ {\frac{{{{\left| {{\Omega _\alpha }\left( {0,{\mathbf{k}}} \right) } \right| }^2}}}{{\left( {{\gamma _a} + i\left( {ck - {\omega _g}} \right) } \right) }}} \right] S\left( 0 \right) \end{aligned}$$

In order to simplify this expression, we introduce local density of states (LDOS), $\rho \left( \omega \right)$, such that $\rho \left( \omega \right) d\omega$ gives the number of modes within the frequency interval $\omega$ to $\omega + d\omega$^54^. This enables us to write the integral from Eq. (19) as

20 $$\begin{aligned} \sum \limits _\alpha {\int {\frac{{{d^3}{\mathbf{k}}}}{{{{\left( {2\pi } \right) }^3}}}} } \left[ {\frac{{{{\left| {{\Omega _\alpha }\left( {0,{\mathbf{k}}} \right) } \right| }^2}}}{{\left( {{\gamma _a} + i\left( {ck - {\omega _g}} \right) } \right) }}} \right] = \int {\frac{{d\omega \,\rho \left( \omega \right) {{{\tilde{\Omega }} }^2}\left( \omega \right) }}{{{\gamma _a} + i\left( {\omega - {\omega _g}} \right) }}} \end{aligned}$$

where ${{\tilde{\Omega }} ^2}\left( \omega \right)$ is the interaction constant, averaged over all possible directions of the wave vector ${\mathbf{k}}$, and over two possible polarizations of light (for details, see^2,54^). It should be noted that both the LDOS and the interaction constant depend on the box size *L*, however, their product, ${{\tilde{\Omega }} ^2}\left( \omega \right) \rho \left( \omega \right)$, does not. We then use the Sokhotski–Plemelj theorem^55^ to calculate the integral in Eq. (19) in the limit ${\gamma _a} \rightarrow 0$. After combining Eqs. (19) and (20) and employing the Sokhotski–Plemelj theorem, we arrive at:

21 $$\begin{aligned} {\gamma _\sigma } + i\left( {{\omega _{TLS}} - {\omega _g}} \right) = - i{D_0}N\int {d\omega \,\rho \left( \omega \right) {{{\tilde{\Omega }} }^2}\left( \omega \right) \left( {1/\left( {\omega - {\omega _g}} \right) + i\pi \delta \left( {\omega - {\omega _g}} \right) } \right) } \end{aligned}$$

where the frequency integral is determined in terms of the Cauchy principal value. Now, in Eq. (21), we separate the real and imaginary parts to obtain the system of equations:

22 $$\begin{aligned} {\gamma _\sigma }= & {} {D_0}N\pi \,\rho \left( {{\omega _g}} \right) \,{{\tilde{\Omega }} ^2}\left( {{\omega _g}} \right) \end{aligned}$$

23 $$\begin{aligned} {\omega _{TLS}} - {\omega _g} = - {D_0}N\int {\frac{{\rho \left( \omega \right) {{{\tilde{\Omega }} }^2}\left( \omega \right) d\omega }}{{\omega - {\omega _g}}}} \end{aligned}$$

Equations (22)–(23) can be simplified further. The expressions on the right-hand side of these equations are proportional to the rates of spontaneous emission^2,48^:

24 $$\begin{aligned} {\gamma _{sp}}\left( {{\omega _g}} \right) = \pi \,\rho \left( {{\omega _g}} \right) \,{{\tilde{\Omega }} ^2}\left( {{\omega _g}} \right) \end{aligned}$$

and a term resembling the Lamb shift^48^:

25 $$\begin{aligned} \Delta \left( {{\omega _g}} \right) = \int {\frac{{\rho \left( \omega \right) {{{\tilde{\Omega }} }^2}\left( \omega \right) d\omega }}{{\omega - {\omega _g}}}} \end{aligned}$$

with the principal difference being its dependence on the lasing frequency ${\omega _g}$, rather than on the transition frequency of an active atom ${\omega _{TLS}}$.

Finally, using Eq. (22), we substitute ${D_0}$ into Eq. (23) and employ Eqs. (24) and (25) to rewrite Eqs. (22) and (23) as:

26 $$\begin{aligned} D_{th}= & {} \frac{{{\gamma _\sigma }}}{{N{\gamma _{sp}}\left( {{\omega _g}} \right) }} \end{aligned}$$

27 $$\begin{aligned} {\omega _g}= & {} {\omega _{TLS}} + \frac{{{\gamma _\sigma }}}{{{\gamma _{sp}}\left( {{\omega _g}} \right) }}\Delta \left( {{\omega _g}} \right) \end{aligned}$$

The expression for the rate of spontaneous emission in Eq. (24) takes into account the Purcell factor^31^ in the location of active atoms.

### Single-mode laser

In this section, we demonstrate that Eqs. (22) and (23) yield the correct lasing frequency and lasing threshold for a single-mode laser. In the single-mode laser we have $\left| {{\omega _{TLS}} - {\omega _{CM}}} \right|< < {\omega _{CM}}$, where ${\omega _{CM}}$ is a frequency of cavity mode^2^. Our aim is to evaluate the integral from Eq. (23):

28 $$\begin{aligned} \int \limits _0^\infty {f\left( {\omega ,{\omega _g}} \right) \,} d\omega = V\int \limits _0^\infty {\frac{{\rho \left( \omega \right) \Omega _{cav}^2}}{{\omega - {\omega _g}}}} d\omega \end{aligned}$$

where $\Omega _{cav}^2$ is an interacting constant between the cavity mode and the active atoms.

We use the expression for the DOS in a single-mode lossy cavity, $V\rho \left( \omega \right) = \frac{{{\gamma _{CM}}/\pi }}{{{{\left( {\omega - {\omega _{CM}}} \right) }^2} + \gamma _{CM}^2}}$^2^. We extend the integration limits in (18) from $\left( {0,\, + \infty } \right)$ to $\left( { - \infty ,\, + \infty } \right)$ to enable the use of the residue theorem. This transition is justified because the expression under the integral in Eq. (28) has a shape of Lorentz curve, therefore, the value of the integral is mostly determined by the interval where the denominator is close to zero, i.e. near the points $\omega = {\omega _{CM}}$ and $\omega = {\omega _g}$. Thus, we choose a contour lying in the half-plane, ${\mathop {\mathrm{Im}}\nolimits } \omega > 0$, and write

29 $$\begin{aligned} \int \limits _{ - \infty }^\infty {f\left( {\omega ,{\omega _g}} \right) \,} d\omega = 2\pi i \times {\mathrm{Re}}{{\mathrm{s}}_{\mathrm{1}}} + \pi i \times {\mathrm{Re}}{{\mathrm{s}}_{\mathrm{2}}} \end{aligned}$$

where ${\mathrm{Re}}{{\mathrm{s}}_{\mathrm{1}}}$ is the residue at a point $\omega = {\omega _{CM}} + i{\gamma _a}$ in the complex plain and ${\mathrm{Re}}{{\mathrm{s}}_{\mathrm{2}}}$ is the residue at a point $\omega = {\omega _g}$. After algebraic operations, we obtain

30 $$\begin{aligned} \int \limits _0^\infty {f\left( {\omega ,{\omega _g}} \right) \,} d\omega \approx \int \limits _{ - \infty }^\infty {f\left( {\omega ,{\omega _g}} \right) \,} d\omega = \Omega _{cav}^2\frac{{{\omega _{CM}} - {\omega _g}}}{{{{\left( {{\omega _{CM}} - {\omega _g}} \right) }^2} + \gamma _{CM}^2}} \end{aligned}$$

Since we know the expression for the DOS, $V\rho \left( \omega \right)$, we can explicitly write ${\gamma _{sp}}$ as

31 $$\begin{aligned} {\gamma _{sp}}\left( {{\omega _g}} \right) = \pi \,V\rho \left( {{\omega _g}} \right) \,{{\tilde{\Omega }} ^2}\left( {{\omega _g}} \right) = \frac{{{\gamma _{CM}}\,\Omega _{cav}^2}}{{{{\left( {{\omega _g} - {\omega _{CM}}} \right) }^2} + \gamma _{CM}^2}} \end{aligned}$$

Applying Eqs. (28), (30) and (31) to Eq. (21) yields two conditions (derived from the real and imaginary parts of Eq. (21)):

32 $$\begin{aligned} {\gamma _\sigma }\,\Omega _{cav}^2\frac{{{\omega _{CM}} - {\omega _g}}}{{{{\left( {{\omega _{CM}} - {\omega _g}} \right) }^2} + \gamma _{CM}^2}} = {\gamma _{CM}}\,\Omega _{cav}^2\frac{{{\omega _g} - {\omega _{TLS}}}}{{{{\left( {{\omega _g} - {\omega _{TLS}}} \right) }^2} + \gamma _{CM}^2}};\,\,\,\,\,{\gamma _\sigma } = {D_0}N\frac{{{\gamma _{CM}}\,\Omega _{cav}^2}}{{{{\left( {{\omega _{CM}} - {\omega _g}} \right) }^2} + \gamma _{CM}^2}} \end{aligned}$$

The first of these equations yields the well-known formula for the lasing frequency of a single-mode laser (“mode-pulling equation”)^2,20^:

33 $$\begin{aligned} {\omega _g} = \frac{{{\gamma _{CM}}\,{\omega _{TLS}} + {\gamma _\sigma }\,{\omega _{CM}}}}{{{\gamma _{CM}} + {\gamma _\sigma }}} \end{aligned}$$

Inserting the expression from Eq. (33) into the second equation in Eq. (32) enables us to write an expression for threshold population inversion:

34 $$\begin{aligned} {D_{th}} = \frac{{{\gamma _{CM}}\,{\gamma _\sigma }}}{{N\,\Omega _{cav}^2}}\left( {1 + \frac{{{{\left( {{\omega _{CM}} - {\omega _g}} \right) }^2}}}{{\gamma _{CM}^2}}} \right) \end{aligned}$$
